# Statins for the Primary Prevention of Coronary Heart Disease

**DOI:** 10.1155/2019/4870350

**Published:** 2019-01-29

**Authors:** Min Li, Xiaoli Wang, Xinyi Li, Heqing Chen, Yeyin Hu, Xiatian Zhang, Xiaoyi Tang, Yaodong Miao, Guihua Tian, Hongcai Shang

**Affiliations:** ^1^Key laboratory of Chinese Internal Medicine of Ministry of Education and Beijing, Dongzhimen Hospital, Beijing University of Chinese Medicine, Beijing, China; ^2^Beijing University of Chinese Medicine, Beijing, China; ^3^First Teaching Hospital of Tianjin University of Traditional Chinese Medicine, Tianjin, China; ^4^Institute of Integration of Traditional Chinese and Western Medicine, Guangzhou Medical University, Guangzhou, China

## Abstract

**Object:**

The purpose of this study was to fully assess the role of statins in the primary prevention of coronary heart disease (CHD).

**Methods:**

We searched six databases (PubMed, the Cochrane Library, Web of Science, China National Knowledge Infrastructure, Wanfang Database, and Chinese Scientific Journal Database) to identify relevant randomized controlled trials (RCTs) from inception to 31 October 2017. Two review authors independently assessed the methodological quality and analysed the data using Rev Man 5.3 software. Risk ratios and 95% confidence intervals (95% CI) were pooled using fixed/random-effects models. Funnel plots and Begg's test were conducted to assess publication bias. The quality of the evidence was evaluated using the Grading of Recommendations Assessment, Development and Evaluation (GRADE) approach.

**Results:**

Sixteen RCTs with 69159 participants were included in this review. Statins can effectively decrease the occurrence of angina (RR=0.70, 95% CI: 0.58~0.85, I^2^ =0%), nonfatal myocardial infarction (MI) (RR=0.60, 95% CI: 0.51~0.69, I^2^ =14%), fatal MI (RR=0.49, 95% CI: 0.24~0.98, I^2^ =0%), any MI (RR=0.53, 95% CI: 0.42~0.67, I^2^ =0%), any coronary heart events (RR=0.73, 95% CI: 0.68~0.78, I^2^=0%), coronary revascularization (RR=0.66, 95% CI: 0.55~0.78, I^2^ = 0%), and any cardiovascular events (RR=0.77, 95% CI: 0.72~82, I^2^ = 0%). However, based on the current evidence, there were no significant differences in CHD deaths (RR=0.82, 95% CI: 0.66~1.02, I^2^=0%) and all-cause mortality (RR=0.88, 95% CI: 0.76 ~1.01, I^2^ =58%) between the two groups. Additionally, statins were more likely to result in diabetes (RR=1.21, 95% CI: 1.05~1.39, I^2^ =0%). There was no evidence of publication biases, and the quality of the evidence was considered moderate.

**Conclusion:**

Statins seemed to be beneficial for the primary prevention of CHDs but have no effect on CHD death and all-cause mortality.

## 1. Introduction

Cardiovascular diseases (CVDs) are the primary public health problem and a chief cause of morbidity and mortality worldwide. Approximately 17.9 million people die from CVDs every year, accounting for 31% of all deaths globally [1]. Coronary atherosclerotic heart disease, also known as coronary heart disease (CHD), is the largest contributor to CVDs due to atherosclerosis (AS), a chronic inflammatory condition of the coronary arterial wall [2]. AS causes cardiovascular stenosis and/or obstruction, further leading to myocardial ischaemia and hypoxia and ultimately giving rise to myocardial necrosis and even cardiac death. Clinically, CHD is divided into chronic coronary artery disease (stable angina) and acute coronary syndrome (including unstable angina, non-ST-segment elevation myocardial infarction [NSTEMI], ST-segment elevation myocardial infarction [STEMI], and sudden coronary death). CHD causes nearly one-third of all deaths globally [3] and is responsible for 15.5 million persons ≥20 years of age having CHD in the United States [4]. In China, the prevalence of CHD surpassed 80 million in 2010, causing death in over one million people every year [5].

It is well known that CHD is considered a common complex multifactorial disease that may be closely associated with environmental, genetic, and other risk factors, such as hypertension, diabetes mellitus, hyperlipidaemia, cigarette smoking, obesity, and so forth [6, 7]. Many studies have confirmed that controlling risk factors for CHD can effectively reduce cardiovascular events in both symptomatic and asymptomatic individuals [8–10]. In the United States, CHD mortality had been increasing since the 1940s until it reached its peak in approximately 1968. However, in recent decades, the death rate from CHD has dropped sharply and decreased by almost half from 1980 to 2000. The main reason may be due to the control of major risk factors and the increased use of evidence-based medical therapies [8]. Moreover, other countries have observed similar decreases in CHD mortality [9, 10]. These results underscore the enormous value of primary prevention and evidence-based medical treatments in the management of CHD.

There is ample evidence that dyslipidaemia plays a key role in the development and mortality of CHD [11]. Lowering plasma high cholesterol is an important way to reduce the chances of suffering CHD events. Statins, a common type of lipid-lowering drug, have become the first-line therapy for regulating hyperlipidaemia and CHD risk, making them the most widely used prescription drugs around the word [12]. Statins are a potent competitive inhibitor of the 3-hydroxy-3-methylglutaryl coenzyme A (HMG-CoA) reductase, a regulatory enzyme for cholesterol biosynthesis [13]. Pharmacological studies demonstrated that statins can lower total cholesterol (TC) and low density lipoprotein cholesterol (LDL-C) and increase the level of high density lipoprotein cholesterol (HDL-C). Additionally, statins can also inhibit the inflammation reaction, improve endothelial function, and stabilize coronary plaques [14]. Currently, a large number of studies have shown that statins have large secondary prevention effects in patients with CVDs. Simvastatin can decrease the risk of cardiac and all-cause death and the recurrence of myocardial infarction (MI) in patients with CHD [15]. In addition, a systematic review indicated that intensive statin therapy has an excellent effect on lowering the serum lipid level of TC, triglyceride (TG), and LDL-C and on lowering the risk of major adverse cardiac events [16].

However, it is unclear whether statins have similar benefits for individuals without prior CHD. Currently, there are fourteen articles reporting on a similar topic, but most of the studies were associated with primary prevention of CVD. Only two studies were related to CHD, and these two studies were both published in 2000 [17, 18]. In addition, some selection biases can be found in the systematic reviews of primary prevention in CVD. Several studies have focused on elderly patients [19, 20], and some articles have shown that the study participants had diabetes [21, 22]. In addition, a few reviews included trials that partially incorporated patients with a clinical history of CVD [23]. A literature-based meta-analysis showed that statins have limited benefits for all-cause mortality [24], but another study presented the opposite results [23]. All of these findings demonstrate the uncertainty regarding primary prevention of CHD. Thus, the purpose of this study was to reliably determine whether statin therapy can reduce coronary heart events (angina, MI, coronary revascularization, and CHD deaths) among individuals without a history of CHD.

## 2. Methods

This study was performed in accordance with the Preferred Reporting Items for Systematic Reviews and Meta-Analyses (PRISMA) (Supplementary Material 1).

### 2.1. Data Source and Search Strategy

We searched PubMed, the Cochrane Library, Web of Science, China National Knowledge Infrastructure (CNKI), Wanfang Database, and Chinese Scientific Journal Database (VIP) from the inception dates to October 31, 2017. The search strategy used the following general terms individually or combined: “statin”, “HMG-CoA”, “simvastatin”, “fluvastatin”, “lovastatin”, “pravastatin”, “atorvastatin”, “rosuvastatin”, “coronary”, “heart”, “angina”, “CAD”, “CHD”, “myocardial infarct^*∗*^”, “MI”. The detailed search strategy is shown in Supplementary Material 2. We also checked the reference lists of existing reviews to identify the included studies.

### 2.2. Study Inclusion and Exclusion Criteria

We included all randomized controlled trials (RCTs), and the publication language was either English or Chinese. Participants without a clinical history of CHD were included, age and race were not limited. The treatment group was given statins alone or combined with usual care, and the control group was given nothing, placebo, or usual care. Usual care was generally determined based on the specific disease of the participants; for example, patients with diabetes will be given hypoglycaemic agents such as metformin, and patients with hypertension will take captopril or other antihypertensive medicines. If we did not know whether the participants had CHD, these articles were excluded. In addition, we also excluded articles without full text. Moreover, the primary outcomes in this systematic review mainly included angina, nonfatal and/or fatal MI, any coronary heart events, coronary revascularization, and CHD deaths. The secondary outcomes involved any cardiovascular events, CVD deaths and all-cause mortality. We also reported the adverse events, which mainly comprised cancer, diabetes, gastrointestinal/hepatic/renal disorder, myalgia, myopathy, rhabdomyolysis, alanine aminotransferase (ALT), aspartate aminotransferase (AST), creatine kinase (CK), and so forth. The results of the included studies must involve at least one of the primary outcomes.

### 2.3. Data Extraction and Quality Assessment

Two authors (Li, X.Y. and Chen, H.Q.) independently conducted the literature search, study selection, and data extraction. The extracted data of the included studies was entered into a standardized table prepared for this review. The extracted data included the first author, publication year, participant types, sample size, sex, age, interventions in the treatment and control groups, dosage of medications, follow-up time, outcomes, and so on. Disagreements were discussed and resolved at a consensus meeting with the corresponding author. In addition, according to the Cochrane Reviewer's Handbook, the two authors (Hu, Y.Y. and Zhang, X.T.) individually assessed the risk of bias. Six evaluation criteria for the quality of RCTs were used, which included generation of a random sequence, randomization concealment, blinding method, integrity of the outcome data, selective reporting and other bias. Each quality item was graded as low, unclear or high risk.

### 2.4. Statistical Analysis

We used the Rev Man 5.3 software provided by the Cochrane Collaboration to analyse the data [41]. For continuous variables, the outcomes were described as the weighted mean difference (WMD) and 95% confidence interval (95% CI). For dichotomous variables, the data were expressed as risk ratios (RR) with 95% CIs. Means and standard deviations were calculated for continuous variables, and as for the dichotomous data, we recorded the number of patients in each group who suffered the events. Heterogeneity was assessed using the *χ*^2^ test. If I^2^ <50%, the statistical heterogeneity was small, and a fixed-effect model was used for the data analysis. If the I^2^ was more than 50%, we performed the subgroup and sensitivity analyses to determine the reason for the heterogeneity, and a random-effects model was conducted. Descriptive analysis was used if the heterogeneity still existed after subgroup and sensitivity analysis. Potential publication bias was assessed using a funnel plot and Begg's test when there were more than eight trials in a meta-analysis using Stata 14 software [42]. If P>0.05, there is no publication bias; if not, publication bias exists.

### 2.5. GRADE

The GRADE approach was used to evaluate the quality of evidence, which was classified as high, moderate, low, or very low based on judgments regarding the risk of bias, inconsistency, indirectness, imprecision, and other considerations [43]. A summary of the findings (SOF) table was prepared using the software program “GRADE pro GDT”.

## 3. Results

### 3.1. Description of Included Trials

#### 3.1.1. Search Process

**Figure 1:**
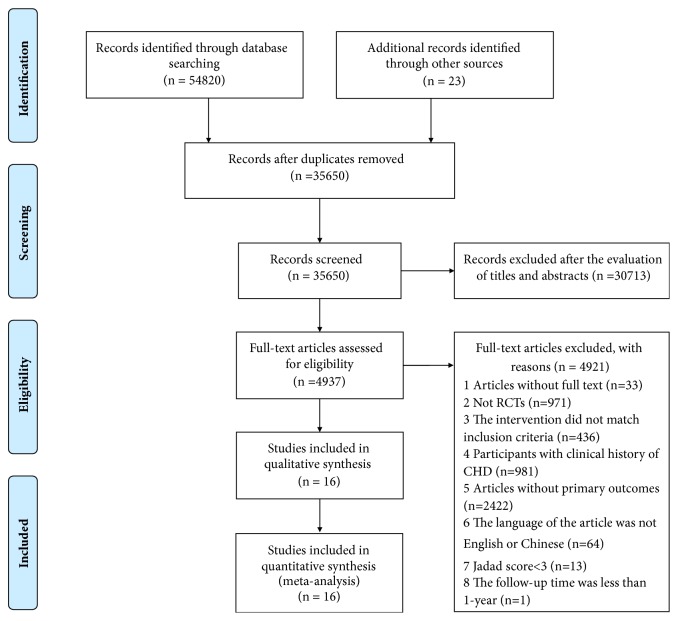
The search process and study selection.

A total of 54820 records were identified by searching the electronic databases, and 23 records were identified from reference lists. After removing 19193 duplicates from the different databases, we evaluated 35650 potentially relevant articles for eligibility. After screening the titles and abstracts, we excluded 30713 studies. Of the 4937 remaining studies, we further excluded 4921 studies after screening the full-text articles. Ultimately, we included 16 studies [25–40]. The search process and study selection process were shown in Figure 1, and the characteristics of the included trials were shown in Table 1.

#### 3.1.2. Participants

A total of 69159 participants with no history of CHD were included, and the numbers of participants in the treatment and control groups were 34582 and 34577, respectively. The average sample size of the trial was 4322 participants. The participants in three studies had hypertension [31, 37, 40], in three studies they had dyslipidaemia [28, 36, 38], in three studies they suffered cerebrovascular diseases [25, 34, 39] and in one study they had type 2 diabetes [27]. In addition, the participants in two articles had carotid atherosclerosis/intima thickening and dyslipidaemia [30, 33]; in four other studies, the participants exhibited aortic stenosis [26], a high level of hs-CRP [32], a high level of hs-CRP/dyslipidaemia [29], and cerebrovascular diseases/diabetes [35].

#### 3.1.3. Interventions

Most studies compared statins and placebo [25–30, 32–35, 37, 38], three articles compared statins plus usual care versus usual care [31, 39, 40], and only one study compared statins plus diet versus diet [36]. Many stains, such as atorvastatin, lovastatin, rosuvastatin, simvastatin, pravastatin, pravastatin sodium, and so forth, were used in the treatment group, and the dosage of the stains ranged from 5 mg/d to 80 mg/d. The control group medications mainly included hydrochlorothiazide, metformin, captopril, amlodipine, and other antihypertensive, hypoglycaemic, and regulating vascular drugs.

#### 3.1.4. Outcomes

All included studies involved at least one of the primary outcomes, nine studies [25, 27–29, 31, 36–39] reported at least one of the secondary outcomes, and seven articles [25, 26, 28, 31, 36–38] reported adverse events.

#### 3.1.5. Follow-Up Time

The shortest follow-up time was 1 year [32, 39, 40], and the longest follow-up time was an average of 5.3 years [36].

#### 3.1.6. Risk of Bias Assessment

**Figure 2:**
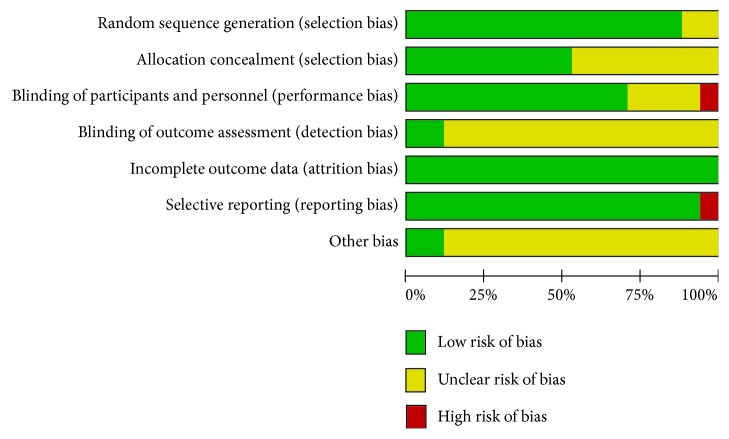
Risk of bias graph.

Fourteen studies generated the random sequence by computer or used a random numbers table [26, 27, 29–40]. Most studies reported that they carried out double-blind analysis [25, 28–30, 33–35, 37–39], and two articles reported that they used a triple-blind method [26, 27]. Two studies reported that the sponsor had no input into the study design and data analysis [26, 37]. The Jadad score of all of the studies was more than 3. The risk of bias assessment is shown in Figure 2.

### 3.2. Effects of Interventions

#### 3.2.1. Primary Outcomes

**Figure 3:**
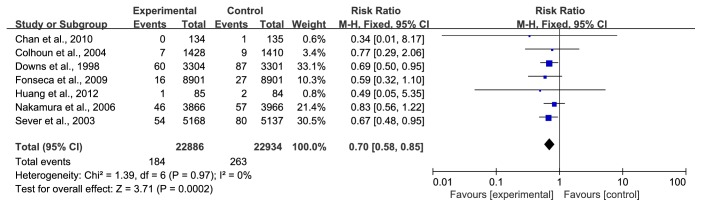
The occurrence of angina pectoris in included studies.

*(1) Angina*. Seven trials [26–29, 32, 36, 37] with 45820 participants, representing 66.25% of the total population, reported angina pectoris. The data was pooled using a fixed-effect model, and no heterogeneity was observed. During the observation period, 184/22886 (0.80%) developed angina in the statin group compared with 263/22934 (1.15%) in the control group. A remarkable difference existed in both groups, and statins exhibited an apparent decrease in the occurrence of angina pectoris (RR=0.70, 95% CI: 0.58~0.85, I^2^ =0%), as shown in Figure 3.

**Figure 4:**
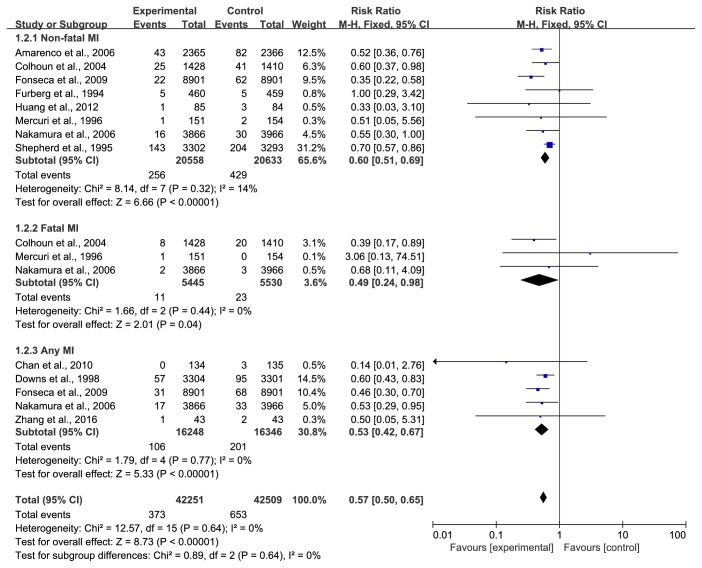
The occurrence of myocardial infarction in included studies.

*(2) Myocardial Infarction*. Three types of MI were described in the included trials, consisting of nonfatal MI, fatal MI and any MI. In the pooled analysis using a fixed-effect model, eight trials [25, 27, 29, 30, 32, 33, 36, 38] with 41191 participants provided strong evidence of a lower recurrence rate of nonfatal MI in the treatment group (RR=0.60, 95% CI: 0.51~0.69, I^2^ =14%). Only three trials [27, 33, 36] with 10975 participants reported fatal MI, and the statin group had a slight advantage over the control group (RR=0.49, 95% CI: 0.24~0.98, I^2^ =0%). In addition, five trials [26, 28, 29, 36, 40], including 19602 men and 12992 women, reported the occurrence of any MI. During the observation period, 106/16248 (0.65%) suffered any MI in the treatment group versus 201/16346 (1.23%) in the control group, and the statin group exhibited a lower occurrence of MI (RR=0.53, 95% CI: 0.42~0.67, I^2^ =0%), as shown in Figure 4.

**Figure 5:**
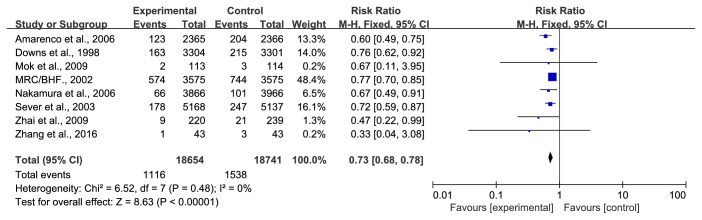
The occurrence of any coronary heart events in included studies.

*(3) Any Coronary Heart Events*. The category any coronary heart events was defined as CHDs, acute coronary syndrome, or other combinations of coronary events (fatal or nonfatal MI, unstable angina, or sudden cardiac death). Eight trials [25, 28, 34–37, 39, 40] with 37395 participants reported any coronary heart events. The duration ranged from 1 year to a mean follow-up of 5.3 years. The data were analysed using a fixed-effect model, and there was no heterogeneity. The prevalence of coronary heart events in the statin group was 5.98% (1116/18654); in the control group, the prevalence was 8.21% (1538/18741); a total of 2678 participants experienced coronary heart events. There was an evident difference in both groups; therefore, statins can apparently reduce the occurrence of any coronary heart events (RR=0.73, 95% CI: 0.68~0.78, I^2^ =0%), as shown in Figure 5.

**Figure 6:**
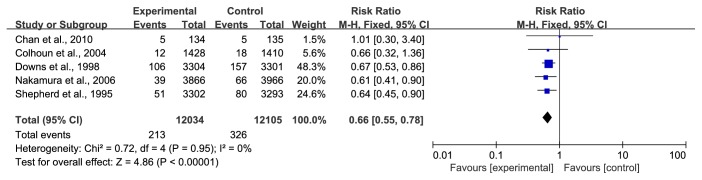
The occurrence of coronary revascularization in included studies.

*(4) Coronary Revascularization*. Thrombolysis, percutaneous coronary intervention (PCI), and coronary artery bypass surgery (CABG) are now collectively known as “coronary revascularization strategy”, which provides symptomatic relief and improves long-term outcomes in patients with CHD. It also reveals the severity of coronary artery disease, exhibiting the number of diseased vessels, the site and degree of coronary obstruction, and the status of collateral circulation. There were five studies [26–28, 36, 38] reflecting coronary revascularization, and compared with the statin group, participants in the control group suffered more coronary revascularization (1.77% versus 2.69%, RR=0.66, 95% CI: 0.55~0.78, I^2^ =0%), as shown in Figure 6.

**Figure 7:**
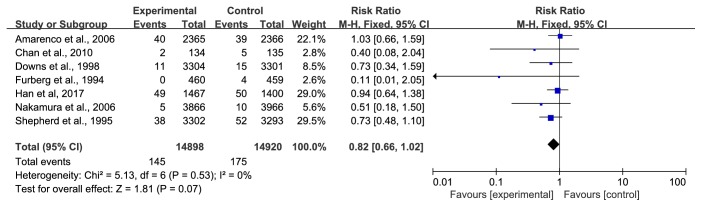
The occurrence of CHD deaths in included studies.

*(5) CHD Deaths*. Seven trials [25, 26, 28, 30, 31, 36, 38] with 29818 participants reported CHD deaths. During the observation period, 145/14898 (0.97%) participants died of CHD in the statin group compared with 175/14920 (1.17%) in the control group. The data were pooled using a fixed-effect model, and no heterogeneity was observed. The results from the meta-analysis demonstrated that there was no significant difference in CHD deaths between the two groups (RR=0.82, 95% CI: 0.66~1.02, I^2^ =0%), as shown in Figure 7.

#### 3.2.2. Secondary Outcomes

**Figure 8:**
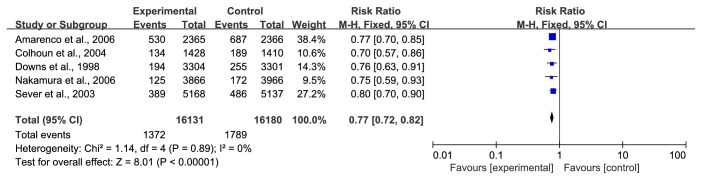
The occurrence of any cardiovascular events in included studies.

*(1) Any Cardiovascular Events*. A total of 3161 participants among a total of 32311 individuals experienced any cardiovascular events in five trials [25, 27, 28, 36, 37]; among them, 1372 cases were in the treatment group and 1789 cases were in the control group. The data were analysed using a fixed-effect model, and there was no heterogeneity. Compared with the control group, the statin group showed that statins can effectively reduce the risk of cardiovascular events (RR=0.77, 95% CI: 0.72~0.82, I^2^ =0%), as shown in Figure 8.

**Figure 9:**
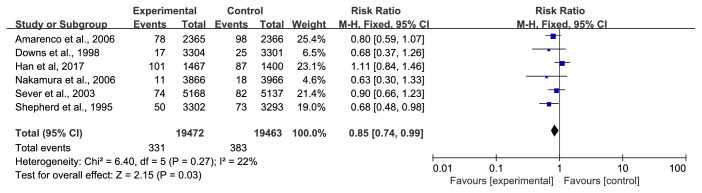
The occurrence of CVD deaths (fixed-effect model).

**Figure 10:**
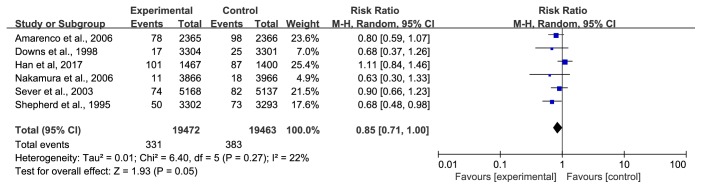
The occurrence of CVD deaths (random-effect model).

*(2) CVD Deaths*. Six trials [25, 28, 31, 36–38] involving 38935 participants, reported CVD deaths. A total of 714 patients suffered CVD deaths, and the numbers of participants in the treatment group and the control group were 331 and 383, respectively. We used a fixed-effect model due to the lower heterogeneity (I^2^ =22%), and the meta-analysis showed that the statin group had a relatively low risk of CVD deaths (RR=0.85, 95% CI: 0.74~0.99) (Figure 9). However, this result changed when we applied a random-effects model, and the 95% CI widened until it reached the ineffective line of forest plots (RR=0.85, 95% CI: 0.71~1.00) (Figure 10). Thus, some findings warrant further discussion.

**Figure 11:**
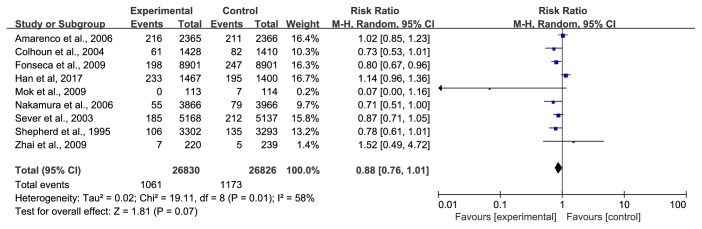
The occurrence of all-cause mortality in included studies.

*(3) All-Cause Mortality*. Nine trials [25, 27, 29, 31, 34–39] with 53656 participants, representing 77.470% of the total population, reported all-cause mortality. The duration ranged from 1 year to a mean follow-up of 5.3 years. The data were pooled using a random-effects model due to relatively greater heterogeneity. During the study period, 1061/26830 (3.95%) died in the statin group compared with 1173/26826 (4.37%) in the control group. The meta-analysis revealed that there was no significant difference in all-cause mortality between the statin group and the control group (RR=0.88, 95% CI: 076~1.01, I^2^ =58%), as shown in Figure 11.

### 3.3. Adverse Events

**Figure 12:**
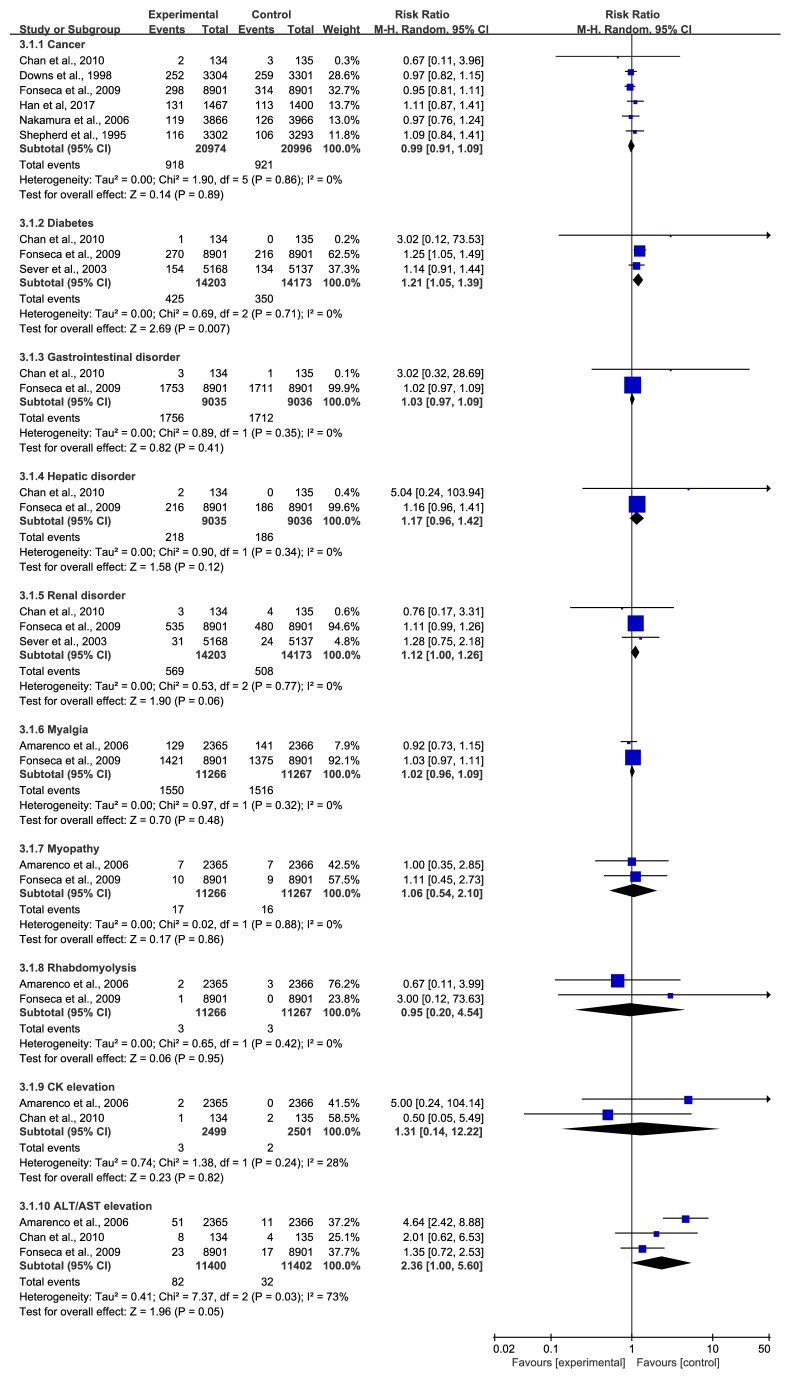
The occurrence of adverse events in included studies.

Several trials described adverse events, including cancer, diabetes mellitus, gastrointestinal/hepatic/renal disorder, myalgia, myopathy, rhabdomyolysis, CK, and ALT/AST. As Figure 12 shows, there were no statistically significant differences in the majority of the adverse events except for the change in diabetes. Three studies [26, 29, 37] reported diabetes mellitus, and the statin group was more prone to this adverse event (RR=1.21, 95% CI: 1.05~1.39, I^2^ =0%). In addition, compared with the control group, the statin group showed a trend toward renal disorder (RR=1.12, 95% CI: 1.00~1.26, I^2^ =0%) and ALT/AST elevation (RR=2.36, 95% CI: 1.00~5.60, I^2^ =73%).

### 3.4. Publication Bias

**Figure 13:**
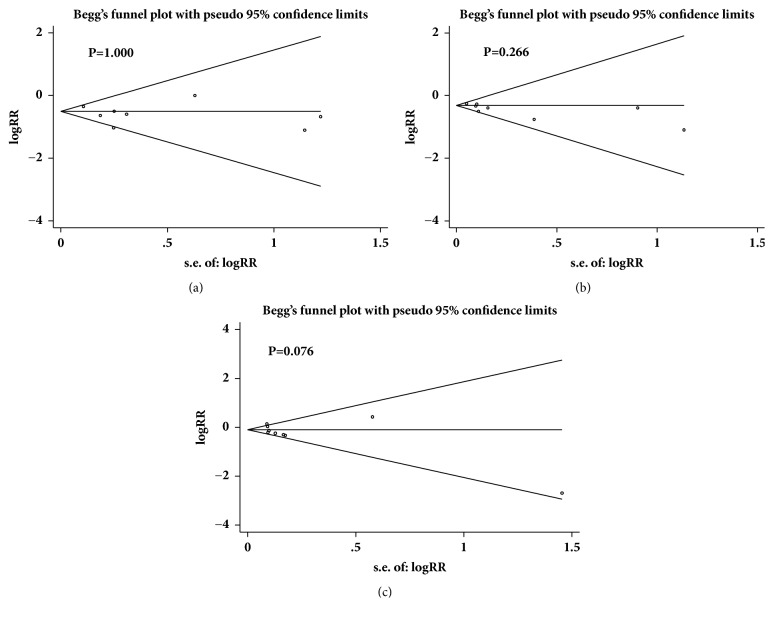
The publication bias of (a): nonfatal MI; (b): any coronary heart events; (c): all-cause mortality.

Potential publication bias was assessed using a funnel plot and Begg's test in Stata 14 software when more than eight trials were included in a meta-analysis. Thus, we evaluated the publication bias for nonfatal MI, any coronary heart events, and all-cause mortality. Funnel plot analysis showed that there was no evidence of publication bias, and Begg's test revealed all P values >0.05, as shown in Figures 13(a)-13(c).

### 3.5. GRADE

The quality of evidence was evaluated by the GRADE approach, and most cardiac events had moderate scores. Some outcomes exhibited low evidence due to wide confidence intervals; the GRADE quality of summary evidence is shown in Table 2.

## 4. Discussion

In these meta-analyses from 16 studies with 69159 participants without a history of CHD, we found that statins can effectively decrease the occurrence of angina, nonfatal and/or fatal MI, any coronary heart events, coronary revascularization and any cardiovascular events. However, based on the current evidence, there were no significant differences in CHD deaths and all-cause mortality between the statin group and control group, and the results for CVD deaths remained controversial. In addition, we analysed the relevant adverse events described in the included studies and found that statin therapy can cause diabetes and increase the trend toward renal disorder and ALT/AST elevation.

Different follow-up time may affect the event outcomes. To verify the effect of follow-up time on the event outcomes, we performed a subgroup analysis of follow-up time. Sixteen RCTs were included in this review. The follow-up time ranged from 1 year to 5.3 years, and the median follow-up was 3.4 years. Thus, we divided the follow-up time into two periods (follow-up <3.4 years; follow-up >3.4 years) for subgroup analysis. We could not perform the subgroup analysis for some primary and secondary outcomes, such as coronary revascularization, CHD deaths, any cardiovascular events and CVD deaths, because no studies or only one study of these outcomes tracked patients for less than 3.4 years. Thus, we conducted a subgroup analysis of the occurrence of angina, nonfatal myocardial infarction, any myocardial infarction, any coronary heart events and all-cause mortality. These results suggested that the length of follow-up may not be a major factor influencing the event outcomes, as shown in Supplemental material 3 (Supplement Figure 1-5). There were two possible reasons for these results. First, in this review, the shortest follow-up was 1 year, while we thought 1 year was a relatively long follow-up time. Second, the follow-up time in this review varied from 1 year to 5.3 years, and we thought that the follow-up gap was not very significant.

As for all-cause mortality in participants without previous CVDs, one study [24] did not find evidence that statin therapy is beneficial in high-risk primary prevention, but the other article [23] reported the opposite results. Our meta-analysis showed that there were no significant differences in CHD deaths and all-cause mortality among participants without a history of CHD, and the conclusion regarding CVD deaths remained controversial. There may be two reasons: on the one hand, statins have a limited impact on mortality; on the other hand, based on current evidence, there is not enough time to observe the death rate. After all, the length of follow-up for this review ranged from 1-year to 5.3 years, and it may take longer to observe participants from no CHD to death.

Many articles have reported that statins exhibit more adverse events [44]. In our review, there was no statistical significance in cancer, gastrointestinal/hepatic/renal disorder, muscular toxicity, and CK elevation between the two groups, but statins exhibited a higher incidence rate of diabetes mellitus. In addition, compared with the control group, the statin group showed a trend toward renal disorder and ALT/AST elevation. These adverse events will cause some patients to stop using statins. However, a cohort study has demonstrated that continued statin prescription after adverse events can lower the incidence of death and cardiovascular events [45]. Thus, the advantages of statins exceed their disadvantages. There are two aspects that may be considered to reduce adverse events. On the one hand, previous studies reported that lipophilic rather than hydrophilic statins easily contributed to cytotoxicity, and this relationship did not correlate with cholesterol-lowering effects [46–48]. Therefore, it is vital to select the appropriate statins according to clinical experience. On the other hand, the study showed that high-dose statins may be beneficial to improve cardiac events, but they also increased the risk of side effects [49]. Nevertheless, some articles presented the opposite conclusion [50, 51]. Thus, a reasonable dose for statins should be chosen based on individual differences.

## 5. Limitations

Most studies did not describe statistical blinding and the role of sponsors in data processing, which was the main methodological omission. In addition, there were not enough studies involving the dose-effect relationship of statins. The participants in the included studies came from America, Canada, China, Japan, the United Kingdom, Ireland and so forth; participants from Africa and South America were lacking. We only included studies in which the language was English or Chinese. In addition, it is a fact that some factors will affect this review's conclusions, such as the inclusion of participants with other diseases (hypertension, dyslipidaemia, cerebrovascular diseases, diabetes, etc.), the use of multiple types and various doses of statins and so forth; all of these easily could have caused inaccuracies in the outcomes.

## 6. Future Directions

Given the available evidence in our work, the results of this review suggested that statins alone or combined with usual care exhibited a specific advantage in the primary prevention of angina and nonfatal and/or fatal MI as well as any coronary heart events. When participants have cardiovascular risk factors, active statin therapy plays a crucial role in preventing the occurrence and improving the prognosis of coronary heart events. For consequent incidental adverse events, it is vital to choose appropriate statins and a reasonable dose of statins based on clinical experience and individual patient differences rather than stop the use of statins. In addition, trials of statins should be performed on all continents around the world to fully reflect the efficacy of statins in all aspects. All studies must report the role of sponsors; after all, this will cause reporting bias.

## 7. Conclusion

Statins seemed beneficial for primary prevention of coronary heart events in participants without evidence of CHD, but there were no statistical differences in CHD deaths and all-cause mortality.

## Figures and Tables

**Table 1:** The characteristics of the included studies.

Study	Participants	Sample (T/C)	Gender (M: F) (T/C)	Age, mean (SD) (T/C)	Intervention (T/C)	Duration	Outcomes
Amarenco et al., 2006 [25]	TIA or stroke	2365/2366	1427:938/1396:970	63.0±0.2/62.5±0.2	Atorvastatin (80 mg/d)/Placebo	4.9 years	②⑤⑦⑧⑨⑩⑪
Chan et al., 2010 [26]	Aortic stenosis	134/135	81:53/85:50	58.0±12.9/57.9±14.3	Rosuvastatin (40 mg/d)/Placebo	3.5 years	①④⑥⑦⑪
Colhoun et al., 2004 [27]	Type 2 diabetes	1428/1410	972:456/957:453	61.5±8.3/61.8±8.0	Atorvastatin (10 mg/d)/Placebo	3.9 years	①②③⑥⑧⑩
Downs et al., 1998 [28]	Dyslipidaemia	3304/3301	2805:499/2803:498	58±7/58±7	Lovastatin (20-40 mg/d)/Placebo	5.2 years	①④⑤⑥⑦⑧⑨⑪
Fonseca et al., 2009 [29]	LDL-C<130 mg/dL and hsCRP≥2.0 mg/L	8901/8901	5475:3426/5526:3375	66.0/66.0	Rosuvastatin (20 mg/d)/Placebo	1.9 years	①②④⑩
Furberg et al., 1994 [30]	Carotid atherosclerosis and Dyslipidaemia	460/459	241:219/232:227	61.80/61.65	Lovastatin (20-40 mg/d)/Placebo	33-month	②⑦
Han et al., 2017 [31]	Hypertension	1467/1400	763:704/689:711	71.3±5.2/71.2±5.2	Pravastatin sodium (40 mg/d)/UC	4.8 years	⑦⑨⑩⑪
Huang et al., 2012 [32]	High level of Hs-CRP	85/84	35:50/36:48	58±4/59±5	Rosuvastatin (5 mg/d)/Placebo	1-year	①②
Mercuri et al., 1996 [33]	Carotid intima thickening and hypercholesterolemia	151/154	85:66/79:75	54.9±5.99/55.1±6.00	Pravastatin (40 mg/d)/Placebo	3 years	②③
Mok et al., 2009 [34]	Asymptomatic middle cerebral artery stenosis	113/114	74:153	63.0±14.0	Simvastatin (20 mg/d)/ Placebo	2 years	⑤⑩
MRC/BHF, 2002 [35]	Diabetes and cerebrovascular disease	3575/3575	Unclear	Unclear	Simvastatin (40 mg/d)/Placebo	5 years	⑤
Nakamura et al., 2006 [36]	Hypercholesterolemia	3866/3966	1528:2338/1248:2718	58.2±7.3/58.4±7.2	Pravastatin (10-20 mg/d) +Diet/Diet	5.3 years	①②③④⑤⑥⑧⑨⑩⑪
Sever et al., 2003 [37]	Hypertension	5168/5137	4189:979/4174:963	63.1±8.5/63.2±8.2	Atorvastatin (10 mg/d)/Placebo	3.3 years	①⑤⑧⑨⑩⑪
Shepherd et al., 1995 [38]	Hypercholesterolemia	3302/3293	All the men	55.3±5.5/55.1±5.5	Pravastatin (40 mg/d)/Placebo	4.9 years	②⑥⑦⑨⑩⑪
Zhai et al., 2009 [39]	TIA	220/239	158:62/169:70	63.2±12.4	Atorvastatin (60 mg/d) + UC/UC	1-year	⑤⑩
Zhang et al., 2016 [40]	Hypertension	43/43	51:35	58.62 ±3.05	Atorvastatin (20 mg/d) + UC/UC	1-year	④⑤

Note: TIA: transient ischaemic attack; LDL-C: low density lipoprotein-cholesterol; hsCRP: hypersensitive C-reactive protein; UC: usual care; ①angina; ②nonfatal MI; ③fatal MI; ④any MI; ⑤coronary heart disease; ⑥coronary revascularization; ⑦CHD deaths; ⑧any cardiovascular event; ⑨CVD deaths; ⑩all-cause mortality; ⑪adverse reactions.

**Table 2:** GRADE quality of evidence summary table.

Outcomes	Illustrative comparative risks^*∗*^ (95% CI)		Relative effect (95% CI)	No. of participants (studies)	Quality of the evidence (GRADE)
	Assumed risk	Corresponding risk			
	Control group	Treatment group			
Angina	Study population		RR 0.7	45820	⊕⊕⊕* *⊝
	11 per 1000	8 per 1000 (7 to 10)	(0.58 to 0.85)	(7 studies)	moderate^1^
	Moderate				
	14 per 1000	10 per 1000 (8 to 12)			
Non-fatal MI	Study population		RR 0.60	41191	⊕⊕⊕* *⊝
	21 per 1000	12 per 1000 (11 to 14)	(0.51 to 0.69)	(8 studies)	moderate^1^
	Moderate				
	21 per 1000	13 per 1000 (11 to 14)			
Fatal MI	Study population		RR 0.49	10975	⊕⊕⊕* *⊝
	4 per 1000	2 per 1000 (1 to 4)	(0.24 to 0.98)	(3 studies)	moderate^1^
	Moderate				
	1 per 1000	0 per 1000 (0 to 1)			
Any MI	Study population		RR 0.53	32594	⊕⊕⊕* *⊝
	12 per 1000	7 per 1000 (5 to 8)	(0.42 to 0.67)	(5 studies)	moderate^1^
	Moderate				
	22 per 1000	12 per 1000 (9 to 15)			
Any coronary heart events	Study population		RR 0.73	37395	⊕⊕⊕* *⊝
	82 per 1000	60 per 1000 (56 to 64)	(0.68 to 0.78)	(8 studies)	moderate^1^
	Moderate				
	67 per 1000	49 per 1000 (46 to 52)			
Coronary revascularization	Study population		RR 0.66	24139	⊕⊕⊕* *⊝
	27 per 1000	18 per 1000 (15 to 21)	(0.55 to 0.78)	(5 studies)	moderate^1^
	Moderate				
	24 per 1000	16 per 1000 (13 to 19)			
CHD deaths	Study population		RR 0.82	29818	⊕⊕⊕* *⊝
	12 per 1000	10 per 1000 (8 to 12)	(0.66 to 1.02)	(7 studies)	moderate^1^
	Moderate				
	16 per 1000	13 per 1000 (11 to 16)			
Any cardiovascular events	Study population		RR 0.77	32311	⊕⊕⊝* *⊝
	111 per 1000	85 per 1000 (80 to 91)	(0.72 to 0.82)	(5 studies)	low^1,2^
	Moderate				
	95 per 1000	73 per 1000 (68 to 78)			
CVD deaths	Study population		RR 0.85	38935	⊕⊕⊕* *⊝
	20 per 1000	17 per 1000 (15 to 19)	(0.74 to 0.99)	(6 studies)	moderate^1^
	Moderate				
	19 per 1000	16 per 1000 (14 to 19)			
All-cause mortality	Study population		RR 0.88	53656	⊕⊕⊕* *⊝
	44 per 1000	38 per 1000 (33 to 44)	(0.76 to 1.01)	(9 studies)	moderate^1^
	Moderate				
	41 per 1000	36 per 1000 (31 to 41)			

^*∗*^ The basis for the assumed risk (e.g., the median control group risk across studies) is provided in footnotes. The corresponding risk (and its 95% confidence interval) is based on the assumed risk in the comparison group and the relative effect of the intervention (and its 95% CI).

CI: confidence interval; RR: Risk ratio.

GRADE Working Group grades of evidence.

High quality: further research is very unlikely to change our confidence in the estimate of effect.

Moderate quality: further research is likely to have an important impact on our confidence in the estimate of effect and may change the estimate.

Low quality: further research is very likely to have an important impact on our confidence in the estimate of effect and is likely to change the estimate.

Very low quality: we are very uncertain about the estimate.

^1^The random sequence generation, allocation concealment, and blinding in some studies were not clear.^2^The confidence interval was wide.

## Data Availability

The data supporting this meta-analysis are from previously reported studies and datasets, which have been cited. The processed data used to support the findings of this study are included within the article.

## Supplementary Materials

Supplementary Materials There were three supplementary materials. The first one is the “PRISMA checklist”, the second one is “The detailed search strategy of included studies”, and the third one is Supplement Figure 1-5: Supplement Figure 1: the occurrence of angina (subgroup analysis). Supplement Figure 2: the occurrence of nonfatal myocardial infarction (subgroup analysis). Supplement Figure 3: the occurrence of any myocardial infarction (subgroup analysis). Supplement Figure 4: the occurrence of any coronary heart events (subgroup analysis). Supplement Figure 5: the occurrence of all-cause mortality (subgroup analysis).

## References

[1] WHO (2017). *World Health Organization. Cardiovascular diseases (CVDs)*.

[2] Infante T., Forte E., Schiano C. (2017). An integrated approach to coronary heart disease diagnosis and clinical management. *American Journal of Translational Research*.

[3] Wong N. D. (2014). Epidemiological studies of CHD and the evolution of preventive cardiology. *Nature Reviews Cardiology*.

[4] Mozaffarian D., Benjamin E. J., Go A. S., Arnett D. K., Blaha M. J., Cushman M. (2016). Heart Disease and Stroke Statistics-2016 Update: A Report from the American Heart Association. *Circulation*.

[5] WHO (2010). World Health Organization. *World Health Statistics Annual*.

[6] Zhou J., Chen X., Ye H. (2014). An Association Study between Genetic Polymorphism in the Interleukin-6 Receptor Gene and Coronary Heart Disease. *BioMed Research International*.

[7] Akhabue E., Thiboutot J., Cheng J.-W. (2014). New and emerging risk factors for coronary heart disease. *The American Journal of the Medical Sciences*.

[8] Ford E. S., Ajani U. A., Croft J. B. (2007). Explaining the decrease in U.S. deaths from coronary disease, 1980–2000. *The New England Journal of Medicine*.

[9] Unal B., Sözmen K., Arık H. (2008). Explaining the decline in coronary heart disease mortality in Turkey between 1995 and 2008. *BMC Public Health*.

[10] Pereira M., Azevedo A., Lunet N. (2013). Explaining the decline in coronary heart disease mortality in Portugal between 1995 and 2008. *Circulation: Cardiovascular Quality and Outcomes*.

[11] Charland S. L., Stanek E. J. (2014). Sigmoidal maximal effect modeling of low-density lipoprotein cholesterol concentration and annual incidence of coronary heart disease events in secondary prevention trials. *Pharmacotherapy*.

[12] Carneiro A. V., Costa J., Borges M. (2004). Statins for primary and secondary prevention of coronary heart disease. A scientific review. *Revista Portuguesa de Cardiologia*.

[13] Istvan E. S., Deisenhofer J. (2001). Structural mechanism for statin inhibition of HMG-CoA reductase. *Science*.

[14] Sirtori C. R. (2014). The pharmacology of statins. *Pharmacological Research*.

[15] Scandinavian Simvastatin Survival Study Group (1994). Randomised trial of cholesterol lowering in 4444 patients with coronary heart disease: the Scandinavian Simvastatin Survival study (4S). *The Lancet*.

[16] Yan Y. L., Qiu B., Hu L. J. (2013). Efficacy and safety evaluation of intensive statin therapy in older patients with coronary heart disease: A systematic review and meta-analysis. *European Journal of Clinical Pharmacology*.

[17] Pignone M., Phillips C., Mulrow C. (2000). Use of lipid lowering drugs for primary prevention of coronary heart disease: Meta-analysis of randomised trials. *British Medical Journal*.

[18] Evans P. H. (2000). The primary prevention of coronary heart disease with statins: practice headache or public health?. *British Journal of General Practice*.

[19] Teng M., Lin L., Zhao Y. J. (2015). Statins for Primary Prevention of Cardiovascular Disease in Elderly Patients: Systematic Review and Meta-Analysis. *Drugs & Aging*.

[20] Lowe R. N., Griend J. P. V., Saseen J. J. (2015). Statins for the primary prevention of cardiovascular disease in the elderly. *Consultant Pharmacist*.

[21] de Vries F. M., Denig P., Pouwels K. B., Postma M. J., Hak E. (2012). Primary prevention of major cardiovascular and cerebrovascular events with statins in diabetic patients: a meta-analysis. *Drugs*.

[22] Chen Y.-H., Feng B., Chen Z.-W. (2012). Statins for primary prevention of cardiovascular and cerebrovascular events in diabetic patients without established cardiovascular diseases: a meta-analysis. *Experimental and Clinical Endocrinology & Diabetes*.

[23] Taylor F., Huffman M. D., Macedo A. F. (2013). Statins for the primary prevention of cardiovascular disease. *Cochrane Database of Systematic Reviews*.

[24] Ray K. K., Seshasai S. R. K., Erqou S. (2010). Statins and all-cause mortality in high-risk primary prevention: A meta-analysis of 11 randomized controlled trials involving 65 229 participants. *JAMA Internal Medicine*.

[25] Amarenco P., Bogousslavsky J., Callahan A. (2006). High-Dose Atorvastatin after Stroke or Transient Ischemic Attack. *The New England Journal of Medicine*.

[26] Chan K. L., Teo K., Dumesnil J. G., Ni A., Tam J. (2010). Effect of lipid lowering with rosuvastatin on progression of aortic stenosis: results of the aortic stenosis progression observation: measuring effects of rosuvastatin (ASTRONOMER) trial. *Circulation*.

[27] Colhoun H. M., Betteridge D. J., Durrington P. N. (2004). Primary prevention of cardiovascular disease with atorvastatin in type 2 diabetes in the Collaborative Atorvastatin Diabetes Study (CARDS): multicentre randomised placebo-controlled trial. *The Lancet*.

[28] Downs J. R., Clearfield M., Weis S. (1998). Primary prevention of acute coronary events with lovastatin in men and women with average cholesterol levels: results of AFCAPS/TexCAPS. *The Journal of the American Medical Association*.

[29] Fonseca F. A. H., Izar M. C. O. (2009). Primary prevention of vascular events in patients with high levels of C-reactive protein: The JUPITER study. *Expert Review of Cardiovascular Therapy*.

[30] Furberg C. D., Adams H. P., Applegate W. B. (1994). Effect of lovastatin on early carotid atherosclerosis and cardiovascular events. Asymptomatic Carotid Artery Progression Study (ACAPS) Research Group. *Circulation*.

[31] Han B. H., Sutin D., Williamson J. D. (2017). Effect of statin treatment vs usual care on primary cardiovascular prevention among older adults: The ALLHAT-LLT randomized clinical trial. *JAMA Internal Medicine*.

[32] Huang G. D., Wang H., Huang X. Z. (2012). Protective effects of low-dose rosuvastatin on high-sensitivity C-reactive protein in the elderly. *New Medicine*.

[33] Mercuri M., Bond M. G., Sirtori C. R. (1996). Pravastatin reduces carotid intima-media thickness progression in an asymptomatic hypercholesterolemic Mediterranean population: The Carotid Atherosclerosis Italian Ultrasound Study. *American Journal of Medicine*.

[34] Mok V. C. T., Lam W. W. M., Chen X. Y. (2009). Statins for asymptomatic middle cerebral artery stenosis: The regression of cerebral artery stenosis study. *Cerebrovascular Disease*.

[35] Heart Protection Study Collaborative Group (2002). MRC/BHF Heart Protection Study of cholesterol lowering with simvastatin in 20* *536 high-risk individuals: a randomised placebocontrolled trial. *The Lancet*.

[36] Nakamura H., Arakawa K., Itakura H. (2006). Primary prevention of cardiovascular disease with pravastatin in Japan (MEGA Study): a prospective randomised controlled trial. *The Lancet*.

[37] Sever P. S., Dahlöf B., Poulter N. R. (2003). Prevention of coronary and stroke events with atorvastatin in hypertensive patients who have average or lower-than-average cholesterol concentrations, in the Anglo-Scandinavian Cardiac Outcomes Trial—Lipid Lowering Arm (ASCOT-LLA): a multicentre randomised controlled trial. *The Lancet*.

[38] Shepherd J., Cobbe S. M., Ford I. (1995). Prevention of coronary heart disease with pravastatin in men with hypercholesterolemia. *The New England Journal of Medicine*.

[39] Zhai Z. Y., Nie Y. X., Li J. S., Zhang C. D. (2009). Effect of Atorvastatin on the Prognosis of Patients with Transient Ischemic Attack. *Herald of Medicine*.

[40] Zhang C. H., Xin T. X. (2016). Effect of Atorvastatin Combined with Irbesartan on pulse pressure and left ventricular hypertrophy in patients with essential hypertension. *Clinical Medicine*.

[41] Higgins J. P. T., Green S. (2011). *Cochrane Handbook for Systematic Reviews of Interventions*.

[42] Begg C. B., Mazumdar M. (1994). Operating characteristics of a rank correlation test for publication bias. *Biometrics*.

[43] Schunemann H., Brozek J., Guyatt G., Oxman A. (2013). GRADE handbook for grading quality of evidence and strength of recommendations. *The GRADE Working Group*.

[44] Bełtowski J., Wójcicka G., Jamroz-Wiśniewska A. (2009). Adverse effects of statins - Mechanisms and consequences. *Current Drug Safety*.

[45] Zhang H., Plutzky J., Shubina M., Turchin A. (2017). Continued statin prescriptions after adverse reactions and patient outcomes: A cohort study. *Annals of Internal Medicine*.

[46] Kobayashi M., Chisaki I., Narumi K. (2008). Association between risk of myopathy and cholesterol-lowering effect: A comparison of all statins. *Life Sciences*.

[47] Taha D. A., Zgair A., Lee J. B. (2017). Hyperlipidaemia alone and in combination with acidosis can increase the incidence and severity of statin-induced myotoxicity. *European Journal of Pharmaceutical Sciences*.

[48] Skottheim I. B., Gedde-Dahl A., Hejazifar S., Hoel K., Åsberg A. (2008). Statin induced myotoxicity: The lactone forms are more potent than the acid forms in human skeletal muscle cells in vitro. *European Journal of Pharmaceutical Sciences*.

[49] Priti K., Agrawal A., Ranwa B. L. (2017). High versus low dose statin therapy in Indian patients with acute ST-segment elevation myocardial infarction undergoing thrombolysis. *Indian Heart Journal*.

[50] Nishikido T., Oyama J.-I., Keida T., Ohira H., Node K. (2016). High-dose statin therapy with rosuvastatin reduces small dense LDL and MDA-LDL: The Standard versus high-dose therApy with Rosuvastatin for lipiD lowering (SARD) trial. *Journal of Cardiology*.

[51] Fang J.-X., Wang E.-Q., Wang W., Liu Y., Cheng G. (2017). The efficacy and safety of high-dose statins in acute phase of ischemic stroke and transient ischemic attack: a systematic review. *Internal and Emergency Medicine*.

