# Supplementary material for: Statins for the Primary Prevention of Coronary Heart Disease
Source: Biomed Res Int. 2019 Jan 29;2019:4870350. doi: 10.1155/2019/4870350 (PMC6374814; doi:10.1155/2019/4870350)
Supplement: Supplementary Materials — There were three supplementary materials. The first one is the “PRISMA checklist”, the second one is “The detailed search strategy of included studies”, and the third one is Supplement Figure 1-5: Supplement Figure 1: the occurrence of angina (subgroup analysis). Supplement Figure 2: the occurrence of nonfatal myocardial infarction (subgroup analysis). Supplement Figure 3: the occurrence of any myocardial infarction (subgroup analysis). Supplement Figure 4: the occurrence of any coronary heart events (subgroup analysis). Supplement Figure 5: the occurrence of all-cause mortality (subgroup analysis). [file 4870350.f1.zip › Supplementary materials_BMRI_2620168.doc]

Supplementary material 1. PRISMA checklist

| **Section/topic** | **#** | **Checklist item** | **Reported on page #** |
| --- | --- | --- | --- |
| **TITLE** | | |  |
| Title | 1 | Identify the report as a systematic review, meta-analysis, or both. | 1 |
| **ABSTRACT** | | |  |
| Structured summary | 2 | Provide a structured summary including, as applicable: background; objectives; data sources; study eligibility criteria, participants, and interventions; study appraisal and synthesis methods; results; limitations; conclusions and implications of key findings; systematic review registration number. | 2 |
| **INTRODUCTION** | | |  |
| Rationale | 3 | Describe the rationale for the review in the context of what is already known. | 3-4 |
| Objectives | 4 | Provide an explicit statement of questions being addressed with reference to participants, interventions, comparisons, outcomes, and study design (PICOS). | 4 |
| **METHODS** | | |  |
| Protocol and registration | 5 | Indicate if a review protocol exists, if and where it can be accessed (e.g., Web address), and, if available, provide registration information including registration number. | 4 |
| Eligibility criteria | 6 | Specify study characteristics (e.g., PICOS, length of follow-up) and report characteristics (e.g., years considered, language, publication status) used as criteria for eligibility, giving rationale. | 4 |
| Information sources | 7 | Describe all information sources (e.g., databases with dates of coverage, contact with study authors to identify additional studies) in the search and date last searched. | 4-5 |
| Search | 8 | Present full electronic search strategy for at least one database, including any limits used, such that it could be repeated. | 4-5 |
| Study selection | 9 | State the process for selecting studies (i.e., screening, eligibility, included in systematic review, and, if applicable, included in the meta-analysis). | 4-5 |
| Data collection process | 10 | Describe method of data extraction from reports (e.g., piloted forms, independently, in duplicate) and any processes for obtaining and confirming data from investigators. | 4-5 |
| Data items | 11 | List and define all variables for which data were sought (e.g., PICOS, funding sources) and any assumptions and simplifications made. | 4-5 |
| Risk of bias in individual studies | 12 | Describe methods used for assessing risk of bias of individual studies (including specification of whether this was done at the study or outcome level), and how this information is to be used in any data synthesis. | 4-5 |
| Summary measures | 13 | State the principal summary measures (e.g., risk ratio, difference in means). | 4-5 |
| Synthesis of results | 14 | Describe the methods of handling data and combining results of studies, if done, including measures of consistency (e.g., I^2^) for each meta-analysis. | 4-5 |

| **Section/topic** | **#** | **Checklist item** | **Reported on page #** |
| --- | --- | --- | --- |
| Risk of bias across studies | 15 | Specify any assessment of risk of bias that may affect the cumulative evidence (e.g., publication bias, selective reporting within studies). | 4-5 |
| Additional analyses | 16 | Describe methods of additional analyses (e.g., sensitivity or subgroup analyses, meta-regression), if done, indicating which were pre-specified. | 4-5 |
| **RESULTS** | | |  |
| Study selection | 17 | Give numbers of studies screened, assessed for eligibility, and included in the review, with reasons for exclusions at each stage, ideally with a flow diagram. | 6-8 |
| Study characteristics | 18 | For each study, present characteristics for which data were extracted (e.g., study size, PICOS, follow-up period) and provide the citations. | 6-8 |
| Risk of bias within studies | 19 | Present data on risk of bias of each study and, if available, any outcome level assessment (see item 12). | 9-13 |
| Results of individual studies | 20 | For all outcomes considered (benefits or harms), present, for each study: (a) simple summary data for each intervention group (b) effect estimates and confidence intervals, ideally with a forest plot. | 9-13 |
| Synthesis of results | 21 | Present results of each meta-analysis done, including confidence intervals and measures of consistency. | 9-13 |
| Risk of bias across studies | 22 | Present results of any assessment of risk of bias across studies (see Item 15). | 9-13 |
| Additional analysis | 23 | Give results of additional analyses, if done (e.g., sensitivity or subgroup analyses, meta-regression [see Item 16]). | 9-13 |
| **DISCUSSION** | | |  |
| Summary of evidence | 24 | Summarize the main findings including the strength of evidence for each main outcome; consider their relevance to key groups (e.g., healthcare providers, users, and policy makers). | 13-15 |
| Limitations | 25 | Discuss limitations at study and outcome level (e.g., risk of bias), and at review-level (e.g., incomplete retrieval of identified research, reporting bias). | 15 |
| Conclusions | 26 | Provide a general interpretation of the results in the context of other evidence, and implications for future research. | 15 |
| **FUNDING** | | |  |
| Funding | 27 | Describe sources of funding for the systematic review and other support (e.g., supply of data); role of funders for the systematic review. | 16 |

Supplementary material 2. The detailed search strategy of included studies

**Pubmed**

**#1** (((("Clinical Trial" [Publication Type]) NOT ("Clinical Trial, Phase I" [Publication Type] OR "Observational Study" [Publication Type]))) OR (("Clinical Trials as Topic"[Mesh]) NOT ("Clinical Trials, Phase I as Topic"[Mesh] OR "Observational Studies as Topic"[Mesh]))) OR random*

**#2**  ((((((((((((((((((((statin*[Title/Abstract]) OR HMG-CoA[Title/Abstract]) OR hydroxymethylglutaryl-coa reductase inhibitor*[Title/Abstract]) OR simvastatin[Title/Abstract]) OR fluvastatin[Title/Abstract]) OR cervistatin[Title/Abstract]) OR lovastatin[Title/Abstract]) OR pravastatin[Title/Abstract]) OR atorvastatin[Title/Abstract]) OR rosuvastatin[Title/Abstract]) OR Lipitor[Title/Abstract]) OR Baycol[Title/Abstract]) OR Lescol[Title/Abstract]) OR Mevcor[Title/Abstract]) OR Altocar[Title/Abstract]) OR Pravacol[Title/Abstract]) OR Lipostat[Title/Abstract]) OR Zocor[Title/Abstract]) OR Crestor[Title/Abstract])) OR "Hydroxymethylglutaryl-CoA Reductase Inhibitors"[Mesh]

**#3** (((((((((((((((((coronary) OR heart) OR CAD) OR CHD) OR acute coronary syndrom*) OR ACS) OR myocard* infarct*) OR MI) OR stenocardia*) OR angina) OR angioplast*) OR stent*) OR endoluminal repair*) OR PCI) OR PTCA) OR atherctom*) OR angor pectoris) OR ischemia

**#4**=**#1**AND**#2**AND**#3**

**Web of Science**

#1 TS= (Hydroxymethylglutaryl-CoA Reductase Inhibitors) *OR* TS= (statin*) *OR* TS

= (HMG-CoA) *OR* TS= (hydroxymethylglutaryl-coa reductase inhibitor*) *OR* TS= (simvastatin)

*OR* TS= (fluvastatin) *OR* TS= (cervistatin)

#2 TS= (lovastatin) *OR* TS= (pravastatin) *OR* TS= (atorvastatin) *OR* TS= (rosuvastatin)

*OR* TS= (Lipitor) *OR* TS= (Baycol) *OR* TS= (Lescol)

#3 TS= (Altocar) *OR* TS= (Pravacol) *OR* TS= (Lipostat) *OR* TS= (Zocor) *OR* TS= (Crestor)

*OR* TS= (Mevcor)

#4 #1 or #2 or #3

#5 TS= (coronary) *OR* TS= (heart) *OR* TS= (CAD) *OR* TS= (CHD) *OR* TS= (acute coronary syndrom*) *OR* TS= (ACS) *OR* TS= (myocard* infarct*)

#6 TS= (MI) *OR* TS= (stenocardia*) *OR* TS= (angina) *OR* TS= (angioplast*) *OR* TS= (stent*) *OR*

 TS= (endoluminal repair*) *OR* TS= (PCI)

#7 TS= (PTCA) *OR* TS= (atherctom*) *OR* TS= (angor pectoris) *OR* TS= (ischemia)

#8 #5 or #6 or #7

#9 TS= (Random*)

#10 #4 and #8 and #9

**Cochrane library**

#1	ACS or myocard* infarct* or MI or stenocardia* or angina (Word variations have been searched)

#2	angioplast* or stent* or endoluminal repair* or PCI or PTCA (Word variations have been searched)

#3	atherctom* or angor pectoris or ischemia (Word variations have been searched)	14562

#4	coronary or heart or CAD or CHD or acute coronary syndrom* (Word variations have been searched)

#5	#1 or #2 or #3 or #4

#6	Hydroxymethylglutaryl-CoA Reductase Inhibitors:ti,ab,kw or statin*:ti,ab,kw or HMG-CoA:ti,ab,kw or hydroxymethylglutaryl-coa reductase inhibitor*:ti,ab,kw or simvastatin:ti,ab,kw (Word variations have been searched)

#7	fluvastatin:ti,ab,kw or cervistatin:ti,ab,kw or lovastatin:ti,ab,kw or pravastatin:ti,ab,kw or atorvastatin:ti,ab,kw (Word variations have been searched)

#8	rosuvastatin:ti,ab,kw or Lipitor:ti,ab,kw or Baycol:ti,ab,kw or Lescol:ti,ab,kw or Mevcor:ti,ab,kw (Word variations have been searched)

#9	Altocar:ti,ab,kw or Pravacol:ti,ab,kw or Liposta:ti,ab,kw or Zocor:ti,ab,kw or Crestor:ti,ab,kw (Word variations have been searched)	4

#10	MeSH descriptor: [Hydroxymethylglutaryl-CoA Reductase Inhibitors] explode all trees

#11	#6 or #7 or #8 or #9 or #10

#12	#5 and #11

#13	random* (Word variations have been searched)

#14	#12 and #13

**China National Knowledge Infrastructure (CNKI)**

SU='他汀' AND FT='冠心病'+' 冠状动脉粥样硬化性心脏病'+'心绞痛'+'心肌梗死'+'心肌梗塞'+'心梗'+'缺血性心脏病'+'缺血性心肌病'+'冠脉病'+'冠状动脉病'+'心肌缺血'+'急性冠状动脉综合征'+'CHD'+'CAD'+'CIS'+'ACS'+'UA'+'NSTEMI'+'STEMI' AND FT=随机

**Wanfang Database**

主题:(他汀) * (冠状动脉粥样硬化性心脏病 + 冠心病 + 心绞痛 + 心肌梗死 + 心肌梗塞 + 心梗 + 缺血性心脏病 + 缺血性心肌病 + 冠脉病 + 冠状动脉病 + 心肌缺血 + 急性冠状动脉综合征 + CHD + CAD + CIS + ACS + UA + NSTEMI+ STEMI) * (随机)

**Chinese Scientific Journal Database (VIP)**

　任意字段=acs 或者 任意字段=ua 或者 任意字段=nstemi 或者 任意字段=stemi 或者 任意字段=心肌缺血 或者 任意字段=急性冠状动脉综合征 或者 任意字段=chd 或者 任意字段=cad 或者 任意字段=cis 或者 任意字段=心梗 或者 任意字段=缺血性心脏病 或者 任意字段= 缺血性心肌病 或者 任意字段=冠脉病 或者 任意字段=冠状动脉病 或者 任意字段=冠状动脉粥样硬化性心脏病 或者 任意字段=冠心病 或者 任意字段=心绞痛 或者 任意字段=心肌梗死 或者 任意字段=心肌梗塞 并且 任意字段=随机 并且 题名或关键词=他汀

Supplementary material 3. Supplement Figure 1-5

Supplement Figure 1. The occurrence of angina (subgroup analysis)

Supplement Figure 2. The occurrence of non-fatal myocardial infarction (subgroup analysis)

Supplement Figure 3. The occurrence of any myocardial infarction (subgroup analysis)

Supplement Figure 4. The occurrence of any coronary heart events (subgroup analysis)

Supplement Figure 5. The occurrence of all-cause mortality (subgroup analysis)
